# How to transport veterinary drugs in insulated boxes to avoid thermal damage by heating or freezing

**DOI:** 10.1186/s12917-017-1058-8

**Published:** 2017-05-25

**Authors:** Johannes Horak, Astrid Haberleitner, Günther Schauberger

**Affiliations:** 1 WG Environmental Health, Division for Physiology and Biophysics, Department for Biomedical Sciences, University of Veterinary Medicine Vienna, Veterinärplatz 1, A 1210 Vienna, Austria; 2 Institute of Atmospheric and Cryospheric Sciences, University of Innsbruck, Innrain 52f, A-6020 Innsbruck, Austria; 3 Institute of Pharmacology and Toxicology, Department for Biomedical Sciences, University of Veterinary Medicine Vienna, Veterinärplatz 1, A 1210 Vienna, Austria

**Keywords:** Storage recommendations, Drug, Thermal conditions, Transport

## Abstract

**Background:**

The transport of veterinary drugs must comply with the general standards for drug storage. Although many vehicles are equipped with active heating and/or cooling devices assuring recommended storage conditions, simple insulated transport boxes are also often used. In this study, measurements for typical transport boxes were performed under laboratory conditions by the use of a climate chamber for a temperature of −20 °C and 45 °C to investigate the impact of box size, insulation material, liquid vs. dry filling products, filling degree and other parameters on the thermal performance of insulated boxes. Model calculations and instructions are presented to predict the retention time of recommended drug storage temperatures.

**Results:**

The measurements and the model calculations showed that the loading of the transport boxes with additional water bottles to increase the heat capacity is appropriate to prolong the retention time of the recommended temperature range of the drugs. Insulated transport boxes are not suitable to store drugs over a period of more than approximately 12 h. For practical use a recipe is presented to measure the thermal properties of a transport box and the related retention time for which the recommended storage temperatures can be assured.

**Conclusions:**

The following principles for drug transportation in vehicles are recommended: (1) Before transfer into boxes, drugs should always be thermally preconditioned (2) Increase the filling degree of the boxes with thermally preconditioned water bottles or re-usable thermal packs will increase the heat capacity. Do not deep-freeze the bottles or packs below 0 °C to avoid drug freezing due to contact. (3) Open the lid of the boxes only to uncase drugs that are immediately needed. (4) The bigger the box and the higher the filling degree, the longer the retention time of the transport box. (5) Wherever possible, place the drug box at a cool site inside the vehicle. (6) The monitoring of the inside temperature of the transport boxes is recommended. By the proper use of such transport boxes the recommended temperatures can be maintained over one working day.

## Background

The transport of veterinary drugs in vehicles must comply with the general standards for drug storage in veterinary dispensaries. Although many veterinary vehicles are equipped with active heating and/or cooling devices (active systems [1]) assuring recommended drug storage conditions, simple insulated transport boxes (passive systems [1]) are also often used in veterinary vehicles [2] as well as in emergency medical service (EMS) vehicles [3]. Such boxes are especially needed for the time when the engine is in non-operating state and the air conditioning of the vehicles is not working (parking time, during consultancy). However, such “simple” transport boxes must assure the maintenance of the appropriate storage temperatures of the drugs kept in them. The thermal properties of the transport boxes, which are exposed to the thermal conditions inside the vehicles, are major factors controlling the retention time for which the recommended storage temperatures can be assured.

The quality of medicines can be negatively affected when subjected to inadequate storage temperatures; thermal degradation and the loss of potency of drugs have been reported following the exceedance of certain temperature thresholds [4–6]. Küppers et al. [7] recommended that temperature-sensitive drugs should be replaced after any temperature stress beyond the limits given by the manufacturers (e.g., 25 °C) and that the drugs should be replaced at least once per year. The inactivation process is described by the Arrhenius equation: the higher the temperature, the higher the degradation of active substances [5, 8–10]. The recommendations distinguish in general between cool (controlled room temperature) and cold storage conditions defined by the ranges 2 °C to 25 °C and 2 °C to 8 °C, respectively [11, 12].

Beside bags [13], although boxes are used to transport and store temperature-sensitive drugs in veterinary vehicles [2, 3, 14], it often remains unclear whether they are appropriate for this purpose. However, to the knowledge of the authors, no systematic investigations on the suitability or practicability of passive storage boxes for drug transport in vehicles have been published so far. Both exceedance of the upper temperature limit and a shortfall of the temperature to below the lower limit (e.g., freezing) must be considered. The discrepancy between the thermal needs and requirements to store drugs and the realities was shown by Haberleitner et al. [2] and Ondrak et al. [14] for veterinary vehicles.

The dynamic behaviour of the inside temperature of the box depends on two main features. (1) The heat flow rate through the walls of the box depends of the temperature difference between inside and outside temperature, the insulation of the box (wall thickness and thermal conductivity), and the area of the box walls. (2) The heat content depends on the mass and the thermal capacity of the filling. The lower the heat flow rate (1) and the higher the heat capacity of the box (2), the slower the change of the inside temperature.

In this study, the thermal performance of typical transport boxes were specifically investigated under laboratory conditions to reveal the impact of box size, insulation material, liquid vs. dry filling products, filling degree and other parameters. For practical use, a recipe is presented to measure the thermal properties of a transport box and the related retention time for which the recommended storage temperatures can be assured. With this simple model it can be assessed whether a certain transport box is appropriate to carry veterinary drugs without exceeding a certain threshold temperature. The objective of this paper is to contribute to the quality assurance of veterinary drug storage especially in vehicles.

## Methods

Six insulated boxes considered typical for drug storage use in veterinary vehicles were investigated under laboratory conditions.

### Transport boxes

The transport boxes differed in the type of insulation material, volume, surface area, wall thickness, and colour (Table 1). With the exception of one box (A), which employed a different insulation technique (vacuum plates), the insulation material was expanded polystyrene with a heat conductivity of *λ* = 0.028 W K^−1^ m^−1^, whereas for a vacuum insulated plate, a value of *λ* = 0.005 W K^−1^ m^−1^ was assumed [15]. A vacuum insulated plate consists of an outer layer of expanded polypropylene foam (EPP) with openings for a vacuum insulated panel which is a form of thermal insulation consisting of a gas-tight enclosure surrounding a rigid core, from which the air has been evacuated [15].

**Table 1:** Properties of the studied transport boxes

Box	Volume *V* [dm^3^]	Outside surface area *A* [m^2^]	Wall thickness *d* [cm]
A	60	1.06	4
B	32	0.60	2
C	44	0.80	4
D	54	0.90	4
E	75	1.10	4
F	10	1.13	14

**Fig. 1:**
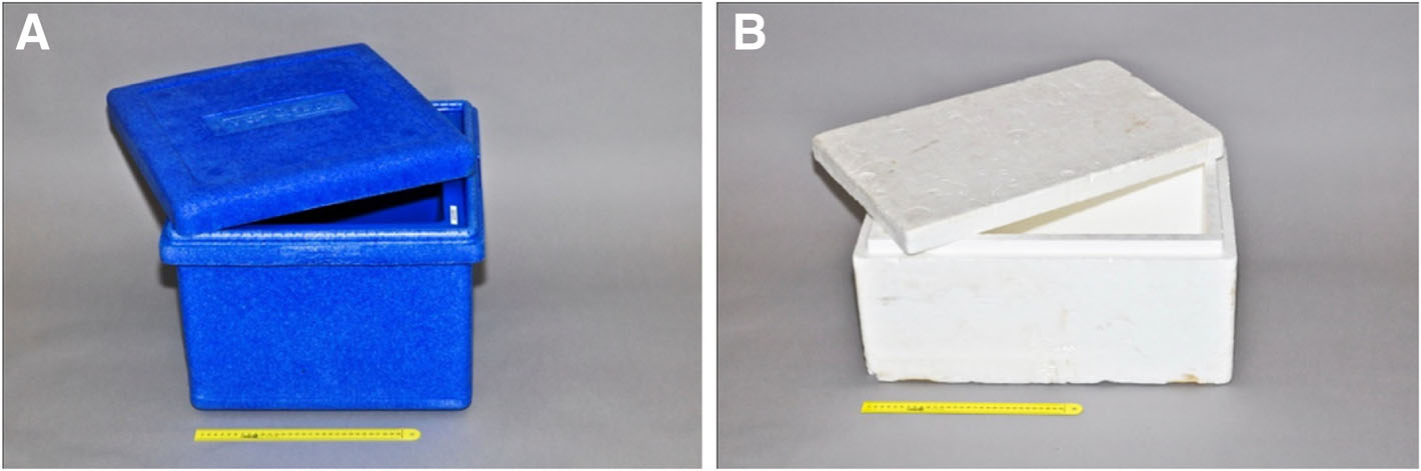
Typical drug transportation boxes. The insulation of box **a** (*left*) consists of vacuum plates, and box **b** (*right*) is insulated only with polystyrene (EPS). The length of the scale is 33 cm

Figure 1 shows two typical transport boxes used in the experiments.

### Measurements

To determine the thermal properties of the boxes, the time courses of the inner temperature of water-filled 100 mL bottles, which simulate injection vials, at defined positions in the box were measured in a heating (45 °C) or cooling chamber (−20 °C) to provide a constant ambient temperature. The contact area of the box to the wall of the climate chamber was minimised to prevent heat exchange due to conduction, by the use of point bearings.

**Fig. 2:**
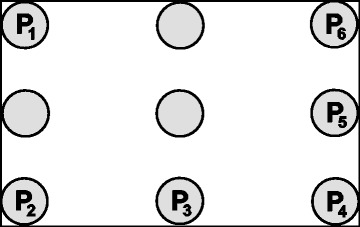
Measurement points inside a transport box. Points *P*_*1*_ to *P*_*6*_ denote the 100 mL water-filled bottles (simulating injection vials) in which the temperature sensors were placed

The temperature was measured at positions P1 to P6 as depicted in Fig. 2. Measurements were performed using a calibrated Fluke Hydra 2620A with 7 channels in 15 s intervals, with channel 7 recording the ambient temperature.

### Determination of the thermal properties of the boxes

The time course of the temperature difference *T*_*B*_ = *Θ*_*B*_ - *Θ*_*A*_ between the inside temperature *Θ*_*B*_ (water bottle) and the ambient temperature *Θ*_*A*_ of the heating chamber (*Θ*_*A*_ = 45 °C) or cooling chamber (*Θ*_*A*_ = −20 °C) is given by the Newton’s law of cooling *T*_*B*_ = *T*_*B* , 0_ exp(−*γ t*) with the temperature difference *T*_*B,0*_ for *t* = 0 [16]. The thermal constant *γ* is given by *γ* = *A U*_*m*_/*c* and specifies the thermal properties of the transport box. This factor was used to characterise the box and depends on its geometry, the overall coefficient of heat transmission *U*_*m*_ (U-value) and the entire heat capacity of its content *c* (transported drugs). The weighted mean of the overall coefficient of heat transmission *U*_*m*_ was calculated with $$ {U}_m=\sum_{i=1}^n{A}_i\;{U}_i/\sum_{i=1}^n{A}_i $$, where *U*_*i*_ is the coefficient of heat transmission of each box wall, and *A*_*i*_ is the corresponding wall surface area. The coefficient of heat transmission of a box wall *U*_*i*_ is given by $$ {U}_i=1/\left(\raisebox{1ex}{$1$}\!\left/ \!\raisebox{-1ex}{${\alpha}_i$}\right.+\sum_{j=0}^n\frac{d_j}{\lambda_j}+\raisebox{1ex}{$1$}\!\left/ \!\raisebox{-1ex}{${\alpha}_o$}\right.\right) $$, where *α*_*i*_ (*α*_*o*_) are the coefficients of heat transfer between the air inside (outside) the box and the adjacent surface. The thickness of a material layer is given by *d*_*j,*_ and its thermal conductivity by *λ*_*j*_. Since there is almost no forced convection inside the box and in the heating or cooling chamber, the value of *α*_*i*_ and *α*_*o*_ can be assumed to be *α*_*i*_ = *α*_*o*_ *= 3* W m^−2^ K^−1^ [17]. The content’s heat capacity is *c* =  ∑ *m*_*i*_ *c*_*i*_ depending on the mass of the drugs *m*_*i*_ and their heat capacity *c*_*i*_.

Following the determination of the time course of the temperature difference *T*_*B*_ during a cooling or heating process, the thermal constant *γ* was determined by a regression analysis. The temperature was measured at the positions depicted in Fig. 2, and the thermal constant *γ*_*i*_ was determined for each of these positions *i*. The maximum value of the thermal constant was selected for further analysis to reflect a worst-case scenario.

The value of *γ* was also calculated using the mechanical and thermal properties given in Table 2. For the calculation of the entire heat capacity of the content (stored drugs) of the boxes, two different types of drugs were distinguished: liquid drugs, which show a high specific heat capacity close to that of water, and dry drugs (e.g., powder, tablets). For dry drugs, the specific heat capacity was estimated to be *c*_*d*_ = 1100 kJ kg^−1^ K^−1^; for liquid drugs (independently if the drugs are hydrophilic or lipophilic), it was estimated to be *c*_*l*_ = 4000 kJ kg^−1^ K^−1^. To increase the heat capacity of the entire box, resulting in a lower thermal constant *γ*, additional water bottles with a volume of 1000 mL were added.

**Table 2:** Mechanical and thermal properties of the materials of the insulated boxes and the drugs inside [15–17]

Mechanical and thermal properties	Symbol	Value
Density (kg m^−3^)		
Glass	*ρ*_*G*_	2600
Air	*ρ*_*A*_	1.2
Water	*ρ*_*W*_	1003
Specific heat capacity (J K^−1^ kg^−1^)		
Glass	*c*_*G*_	750
Air	*c*_*A*_	1000
Water	*c*_*W*_	4190
Drugs (dry) (drugs, blister pack, card)	*c*_*d*_	~1100
Drug (liquid) (drugs, bottle)	*c*_*l*_	~4000
Heat conductivity (W K^−1^ m^−1^)		
Expanded polystyrene (EPS)	λ	0.028
Vacuum plates	λ	0.005
Heat transfer coefficient (W K^−1^ m^−2^)		
Inside and outside	α_i_ and α_o_	3.0

The total heat capacity of a box’s content was calculated as *c* = *m*_*d*_ *c*_*d*_ + *m*_*l*_ *c*_*l*_ + *m*_*w*_ *c*_*w*_ where *m*_*d*_ and *m*_*l*_ denote the mass of dry and liquid drugs (simulated by 100 mL bottles) respectively, and *m*_*w*_ the mass of additional 1000 mL water bottles. The corresponding specific heat capacities are shown in Table 2.

The filling degree *F* was defined as *F* = *c*/*c*_max_with the maximum heat capacity of a box*c*_max_ = *V*_*i*_ *ρ*_*w*_ *c*_*w*_. This assumes that the box’s volume *V*_*i*_ is filled entirely with water (water density *ρ*_*w*_ = 1000 kg m^−3^, and specific heat capacity of water *c*_*w*_ = 4.18 kJ kg^−1^ K^−1^). The heat capacity of the air inside a box was neglected. For the measurements inside the climate chamber, the 100 mL bottles as well as the additional 1000 mL water bottles were preconditioned.

### Model calculations

Two boxes were modelled (Table 3): box X with a volume of 27 dm^3^ (inner dimension 3 × 3 × 3 dm^3^), and box Y with a volume of 54 dm^3^ (inside dimension 6 × 3 × 3 dm^3^). The wall thickness is 5 cm, which results in an outside surface area of 0.96 m^2^ and 1.44 m^2^, respectively. The coefficient of heat transmission was calculated for expanded polystyrene (EPS) with *U*_*m*_ = 0.408 W m ^−2^ K^−1^.

**Table 3:** Parameters for the model calculation for the two transport boxes X and Y

Box properties	Box X	Box Y
Filling volume *V* (dm^3^)	27	54
Surface area *A* (m^2^)	0.96	1.44
Coefficient of heat transmission *U*_*m*_ (W m^−2^ K^−1^) (U-value)	0.408	
Only Drugs (three 100 mL water bottles)		
Heat capacity *c* (kJ K^−1^)	2.28	
Thermal constant *γ* (h^−1^)	0.6184	0.9277
Filling degree *F* (heat capacity) (%)	2	1
Drugs and additional water bottles		
Heat capacity *c* (kJ K^−1^)	102.6	207.1
Thermal constant *γ* (h^−1^)	0.0137	0.0102
Filling degree *F* (heat capacity) (%)	91	92

For the first calculation, the two boxes, X and Y, were filled only with three 100 mL bottles (as used for injectables), and in the second calculation, the remaining volume was filled with 1000 mL water bottles, resulting in a total of 24 to 49 bottles for boxes X and Y respectively. The mass of a full 100 mL bottle totals approximately 190 g. This yielded a heat capacity of 2.28 kJ K^−1^ for the two boxes filled only with drugs and 102.6 kJ K^−1^ for box X and 207.1 kJ K^−1^ for box Y when filled with additional 1000 mL water bottles (Table 3).

## Results

### Measurements

The measurements in the heating (*Θ*_*A*_ = 45 °C) and cooling chamber (*Θ*_*A*_ = −20 °C) were used to determine the thermal constant *γ*_*meas*_ which was then compared to corresponding calculated values *γ*_*calc*_ (Table 4). The calculations were carried out with the mechanical and thermal properties (Table 2) of the transport boxes. A comparison of measured and calculated *γ* values by a regression analyses showed a high agreement with a coefficient of determination of *r* ^*2*^ = 0.843, which is significant at the 0.001 level. The filling degree varied between 4.5% and 17.9%. A mean relative deviation of 22% between *γ*_*meas*_ and *γ*_*calc*_ was found which we attribute to geometric factors (e.g., thermal bridges due to corners, edges and the cover plate). These are not accounted for the chosen model of the coefficient of heat transmission which assumes a plane wall.

**Table 4:** Comparison of the measured *γ*_*meas*_ and the calculated *γ*_*calc*_ thermal constant for the six transport boxes and various relative heat capacities, measured in the heating (30 °C) and/or cooling (−10 °C) chamber

Box	Filling degree *F* [%]	Thermal constant γ [h^−1^]	
		Measured *γ*_*meas*_	Calculated *γ*_*calc*_
A	7.2	0.0256	0.0340
	7.2	0.0319	0.0370
B	3.9	0.2570	0.3090
	9.8	0.1448	0.1200
	13.6	0.1080	0.0870
	13.6	0.0950	0.0870
C	4.6	0.1700	0.1500
	9.9	0.1000	0.0710
D	4.7	0.1250	0.1440
	10.3	0.0670	0.0660
	12.1	0.0711	0.0660
E	4.5	0.1700	0.1300
	8.2	0.1011	0.0730
	10.7	0.1080	0.0660
F	17.9	0.0702	0.0800

### Model calculations

Model calculations were performed to demonstrate the impact of box size and their degree of filling on thermal performance for the two boxes X and Y (Table 3).

**Fig. 3:**
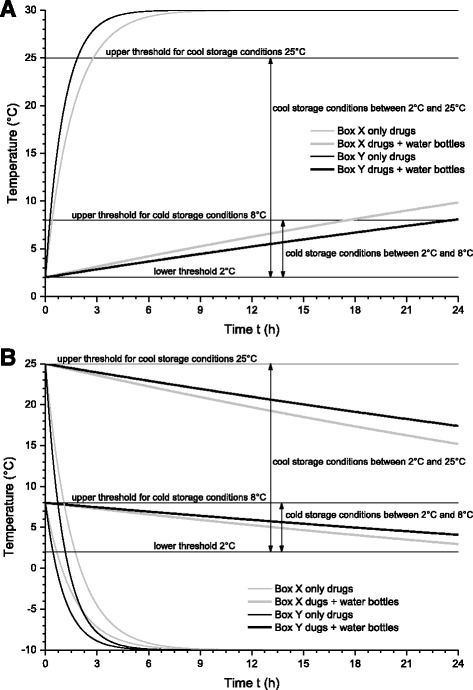
Model calculation of the time course of the inside temperature *Θ*_*B*_ of the two virtual boxes X and Y depending on the size and the filling degree (heat capacity) of the boxes. The chosen parameters represent a summer (**a**) and a winter scenario (**b**) for drugs that must be stored either in a cool (2 °C ≤ *Θ*_*B*_ ≤ 25 °C) or cold environment (2 °C ≤ *Θ*_*B*_ ≤ 8 °C). For the summer scenario, the environmental temperature was *Θ*_*A*_ *=* 30 °C and the initial temperature *Θ*_*B*_ *=* 2 °C at *t* = 0 h. For the winter scenario, the environmental temperature was *Θ*_*A*_ *= −*10 °C, and the initial temperature (*t* = 0 h), depending on the storage conditions (*cool or cold*), was *Θ*_*B*_ *=* 25 °C and *Θ*_*B*_ *=* 8 °C, respectively

These calculations were performed for two different environmental conditions: Summer with an ambient temperature of *Θ*_*A*_ *=* 30 °C and winter with *Θ*_*A*_ *= −*10 °C. The retention time, defined as the time period during which the storage requirements are fulfilled, was then calculated for the two boxes under the assumption that the box content was thermally preconditioned. For summer calculations it was assumed to have an initial temperature of 2 °C, equal to the lower threshold of cold and cool storage conditions. For winter the corresponding upper thresholds where chosen yielding initial temperatures of 8 °C and 25 °C respectively. The resulting time courses of the drug’s temperatures are plotted in Fig. 3, for a (A) summer scenario and (B) winter scenario.

Retention time calculation and various filling degrees for the two boxes are summarised in Table 5. Values below 12 h were marked in bold to show possible drug storage problems during a working day. These calculations show the high impact of the filling degree on retention time, especially for cold storage conditions (2 °C and 8 °C).

**Table 5:** Retention time (h) of the inside temperature in the recommended temperature range (cool storage conditions 2 °C – 25 °C and cold storage conditions 2 °C – 8 °C) in the insulated boxes X and Y for thermal summer and winter conditions with an ambient temperature of *Θ*_*A*_ *=* 30 °C and *Θ*_*A*_ *=* 30 °C, respectively

Box X										
No of additional 1000 mL water bottles	0	1	2	4	6	10	14	15	20	24
Heat capacity of the additional water bottles *c*_*w*_ (kJ K^−1^)	0.0	4.2	8.4	16.7	25.1	41.8	58.5	62.7	83.6	100.3
Entire heat capacity *c* (kJ K^−1^)	2.3	6.5	10.6	19.0	27.4	44.1	60.8	65.0	85.9	102.6
Filling degree *F* (%)	2	6	9	17	24	39	54	58	76	91
Thermal constant *γ* (h^−1^)	0.6184	0.2183	0.1325	0.0742	0.0515	0.0320	0.0232	0.0217	0.0164	0.0137
	Retention time of the inside temperature in the recommended range (h)									
Winter cool 2 °C – 25 °C	**1.7**	**4.9**	**8.1**	14.4	20.8	33.5	46.2	49.3	65.2	77.9
Winter cold 2 °C – 8 °C	**0.7**	**1.9**	**3.1**	**5.5**	**7.9**	12.7	17.5	18.7	24.7	29.5
Summer cool 2 °C – 25 °C	**2.8**	**7.9**	13.0	23.2	33.4	53.9	74.3	79.4	104.9	125.4
Summer cold 2 °C – 8 °C	**0.4**	**1.1**	**1.8**	**3.2**	**4.7**	**7.5**	**10.4**	**11.1**	14.7	17.5
Box Y										
No of additional 1000 mL water bottles	0	1	2	4	6	10	20	30	40	49
Heat capacity of the additional water bottles *c*_*w*_ (kJ K^−1^)	0.0	4.2	8.4	16.7	25.1	41.8	83.6	125.4	167.2	204.8
Entire heat capacity *c* (kJ K^−1^)	2.3	6.5	10.6	19.0	27.4	44.1	85.9	127.7	169.5	207.1
Filling degree *F* (%)	1	3	5	9	13	21	41	61	81	99
Thermal constant *γ* (h^−1^)	0.9277	0.3274	0.1988	0.1113	0.0773	0.0480	0.0246	0.0166	0.0125	0.0102
	Retention time of the inside temperature in the recommended range (h)									
Winter cool 2 °C – 25 °C	**1.2**	**3.3**	**5.4**	**9.6**	13.8	22.3	43.5	64.6	85.8	104.8
Winter cold 2 °C – 8 °C	**0.4**	**1.2**	**2.0**	**3.6**	**5.2**	**8.5**	16.5	24.5	32.5	39.7
Summer cool 2 °C – 25 °C	**1.9**	**5.3**	**8.7**	15.5	22.3	35.9	70.0	104.0	138.0	168.7
Summer cold 2 °C – 8 °C	**0.3**	**0.7**	**1.2**	**2.2**	**3.1**	**5.0**	**9.8**	14.6	19.3	23.6

The drugs and the water bottles were preconditioned for summer and winter conditions at a temperature at the lower (2 °C) and upper (8 °C or 25 °C) limit of the recommended temperature range. Values below a retention time of 12 h were marked in bold

Calculations for boxes X and Y also demonstrate the influence of a box’s size. The ratio of surface area (responsible for the heat exchange between box and environment) to volume decreases with box size. For the same degree of filling *F*, the retention time is greater for the larger box, Y, as compared to the smaller box, X (Table 5).

Due to the fact that Table 5 represents model calculations we added high filling degrees to show the importance of this parameter for the retention time, even if these cannot be reached in reality. This demonstrates that the filling degree *F* should be as big as possible to maximise the retention time of a transport box.

## Discussion

The proper transport of temperature-sensitive drugs in vehicles is a widespread and notorious problem [18–21]. For veterinary vehicles a one year field study was published recently by Haberleitner et al. [2] showing that the drugs are frequently exposed to temperatures not compliant with recommended storage conditions. In many vehicles, drugs are stored in boxes without an active cooling or heating supply. Field measurements showed transport temperatures for emergency medical technician (EMS) vehicles far outside of the recommended temperature range, as summarised by Brown et al. [20]. For summer conditions, maximum inner temperatures up to 60 °C were determined, and for wintertime, minimum temperatures significantly below the freezing point [18, 21]. It became evident that either active heating or cooling systems should be used [18, 22] or other efforts made to maintain the recommended temperatures when passive storage systems are used. However, to the knowledge of the authors, no systematic investigations on the suitability or practicability of passive storage boxes for drug transport in vehicles have been published so far.

Storage temperatures outside the recommended range can affect the quality of drugs [6]. Exceedance of the upper limit of the recommended temperature range may cause a loss of drug quality due to degradation processes. The inactivation process is described by the Arrhenius equation, the higher the temperature the higher the degradation of active substances using the mean kinetic temperature [5, 8–10]. The mandatory shelf life of a temperature-sensitive drug allows for a degradation of active substances of less than 5% during correct storage [7, 9, 23]. For a typical drug, an increase in the mean kinetic temperature of 5 °C will decrease the drug’s shelf life by a factor of two [21]. For the US the storage standards of the US Pharmacopeial Convention are summarised by Brown and Campagna [24].

The lower limit of 2 °C for the storage temperature was selected in this study to avoid freezing the drugs, even though the freezing point is an individual constant of each drug and may differ over a wide range [7]. In particular, injection preparations and vaccines are sensitive to freezing and lose quality and efficacy after thawing. Because “frozen” and “not frozen” distinguish between “(potentially) usable” and “not usable” [7, 12], we selected this limit of 2 °C for pragmatic reasons.

To investigate the practicability of insulated boxes for storing drugs in vehicles, we measured the retention time for winter conditions and summer conditions with an experimental ambient temperature of −10 °C and 30 °C, respectively. The measuring protocol of the World Health Organisation [25] for vaccines suggested the ambient temperatures of −5 °C and 43 °C, respectively. The filling degree of the transport boxes, which describes the heat capacity load, was defined in a similar way as proposed by the German [26] and French [27] standards. The measuring points inside the boxes were selected according to the World Health Organisation [25], and the most adverse measuring point was selected for the calculation [28].

The results obtained from the experiments were compared with the values of the thermal constant *γ* obtained from the model calculation (Table 4). The high coefficient of determination *r* ^*2*^ showed that the selected exponential model corresponds well with the measured time course data for the inner temperature of the box and demonstrates that the thermal constant of practically any transport box can analogously be determined. Thus, the time course and the retention time of any box can be predicted by its geometry, the thermal properties, and the heat capacity of the box as determined by the drugs and the additional water bottles.

Model calculations were performed for two boxes that differ significantly in size and volume to show the influence of these parameters. The time course of the inner temperature of the two boxes differs only slightly (by the constant filling degree of the volume) because the dominant factor is the entire heat capacity of a box. By adding 1000 mL water bottles, the filling degree *F* can be increased up to 90%, and the retention time can be increased by a factor of approximately 45 and 80 for box X and box Y, respectively. This shows the dominant influence of the filling degree on the retention time [25–27]. Insulated transport boxes cannot be considered appropriate to store drugs over a period of more than 12 h (Table 5). This means that after a “working day”, the content of the transport boxes must be stored under appropriate conditions (e.g., transfer into a refrigerator) and thermally preconditioned according to the recommended temperatures before it is transferred back into the box for the next tour. A Haberleitner, G Schauberger, J Horak and I Schmerold [2] were able to show that a proper management of the storage of drugs by using solely styrofoam transport boxes can guarantee the requirements to the thermal storage conditions.

The ambient temperature of the transport boxes is the inside temperature of the vehicles being itself predominantly subjected to the outdoor temperature and the radiation balance if no additional heating and cooling (air-conditioning) is in operation. As long as the incoming solar radiation and the outgoing long-wave radiation is nearly balanced, the indoor temperature of the vehicles is close to the ambient temperature (e.g., during an overcast sky, under a car port, or inside a garage).

If the radiation balance is dominated by the incoming solar radiation, the temperature inside the car stabilises within a range between 20 and 35 K above the outside temperature [29–36]. Marty et al. [37] found a temperature difference even close to 60 K. As a rough estimation, they assessed the inside temperature due to solar radiation could reach 30 °C for winter time, 60 °C for spring and autumn and up to 90 °C during summer time. The harsh environment inside parked vehicles can cause heat stroke as a life-threatening syndrome observed in human and animals [38, 39] which is documented in the US, by 37 lethal heat stokes by children per year (1998–2015) [33, 40].

Grundstein et al. [31] developed simple models to calculate the inside steady state temperature of the cabin as a function of the outside air temperature, the irradiance of the solar radiation, and the cloud cover. The dynamic behaviour of the temperature increase after parking a vehicle in the sun was also measured by several authors [29, 30, 32–34, 36]. A dynamic model for the cabin temperature, which is driven by outside air temperature, solar radiation and wind velocity was presented recently [41]. In most cases, a value close to the maximum temperature is achieved 20 min after stopping the ventilation. The most effective measure to reduce the inside temperature is to increase the ventilation by partly opened windows [32, 34–36]. The protection of the windows by a cover to reduce the incoming solar radiation was investigated by Jascha and Keck [36] (paper fabrics and tin foil) and Devonshire and Sayer [42] using infrared reflecting foils. They found a reduction up to 11 K [36] and a higher score of thermal comfort [42], compared to the unprotected windows. Veterinary vehicles should, therefore, always be parked during daytime (solar radiation) in the shade. The direct exposure of the transport box to solar radiation through car windows should be strictly avoided during summer conditions. The additional heat flow rate could reach up to 1000 W/m^2^. This additional heat load would reduce the retention time of a transport box drastically. Therefore the box should be stored also inside the cabin in the shade.

The use of passive boxes for drug transportation should only be a temporary substitute for air conditioning of the cabin to avoid heating as well as freezing. Estimation and knowledge of the thermal properties of the boxes used is a crucial factor for appropriate drug transport in vehicles and an important contribution to quality assurance in veterinary practice.

## Conclusion

Observing the physical rules governing the thermal performance of passive boxes, the following principles for drug transportation in vehicles can be recommended:

- Before transfer into boxes, the drugs should always be thermally preconditioned (i) at the upper limit of the recommended temperature range for ambient temperatures below the recommended temperature (“winter”) or (ii) at the lower limit for ambient conditions above the recommended temperature (“summer”). The most critical storage condition is the range between 2 °C and 8 °C.
- Increase the filling degree of the box as much as possible by thermally preconditioned water bottles or re-usable thermal packs to increase the heat capacity of the transport box. Do not deep-freeze the bottles or packs below 0 °C to avoid drug freezing due to contact. This recommendation applies also to active devices (e.g., refrigerators).
- Open the lid of the boxes only to uncase needed drugs. Avoid air exchange especially due to wind effects and due to the thermal rise of the warmer air from inside the boxes during cold outside conditions.
- The bigger the box and the higher the filling degree, the longer the retention time of the transport box.
- Wherever possible, place the drug box at a cool site. The vehicle should always be parked in the shade or, if possible, inside a well ventilated car port. The transport box should not be exposed to direct solar radiation.
- The monitoring of the inside temperature of the transport boxes is recommended using remote temperature sensors and/or temperature data loggers to avoid and/or detect violation of recommended storage temperatures.

## Appendix

### Recipe to measure thermal properties of a box and to calculate the retention time

For practical use, a recipe is described to calculate the retention time, for a transport box to avoid an exceedance of a temperature threshold. The recipe includes two steps: (1) measurement of the thermal properties of the box, and (2) calculation of the retention time.

In a first step the thermal constant γ of a selected transport box is measured. The box is filled in the same way as it is proposed for practical use. For the application of the transport box the retention time is calculated for a certain ambient temperature. This retention time gives the reliability that the storage temperature of the transported drugs will not exceed the recommended temperature range.

### Step 1: Measurement of the thermal properties of a box

For the measurements and the calculation the following equipment is required (1) the transport box, (2) water bottles, to simulate the expected filling degree of the box, (3) thermometer (unit: degree Celsius), and (4) calculator. Bottles of arbitrary size are to be filled with water. The mass of water should equal the mass of the pharmaceuticals that are usually transported inside the box at hand. The filled bottles should be cooled in a refrigerator to about 4 °C. It is crucial that none of the water is frozen.

After the cooldown to the preconditioned temperature, the initial box (water) temperature *Θ*_*B,i*_ of at least one of the bottles is measured. Afterwards the box that is to be tested is loaded with the cooled water bottles.

The box should now be placed in a room that fulfils the following requirements (1) low temperature fluctuation, (2) no direct solar radiation, and (3) no draught. The box should stay about 1 m away from corners or walls and the ambient room temperature *Θ*_*A*_ has to be measured.

After about 8 h (depending on the filling degree (Table 5)), the final box (water) temperature *Θ*_*B,f*_ has to be measured. If the difference between *Θ*_*B,f*_ and *Θ*_*B,i*_ is at least 5 °C the experiment is finished, otherwise the duration of 8 h has to be expanded and a new value of the final box temperature *Θ*_*B,f*_ has to be measured. The time *t* is the duration that the box was left in the room in hours. All four parameters *Θ*_*B,i*_, *Θ*_*B,f*_, *Θ*_*A*_, and *t*, which have to be measured, are listed in the highlighted Box of Step 1. The measurement procedure in short: (1) Measure the initial temperature inside the box *Θ*_*B,i*_, (2) leave the box for several hours in a room with constant ambient temperature *Θ*_*A*_, (3) measure the final temperature inside the box *Θ*_*B,f*_, and (4) determine the duration for the measurements inside the room *t*.

These temperatures and the duration of the experiment are inserted into the following equation:

$$ {\gamma}_C=\left| \ln \left(\frac{\Theta_{B, f}-{\Theta}_A}{\Theta_{B, i}-{\Theta}_A}\right)\right|\cdot \frac{1}{t} $$

This yields the constant *γ*_*C*_ which characterises the thermal properties of the investigated box. To be on the safe side, a correction factor *κ =* 1.45 has to be applied which yields

$$ \gamma =\kappa\;{\gamma}_C $$

**Step 1: Measurement of the thermal properties of a box**

**Table:**

**Measurement of the four parameters**	
Initial temperature	*Θ*_*B,i*_ = 5°C
Final temperature	*Θ*_*B,f*_ = 10°C
Mean ambient temperature	*Θ*_*A*_ *= 25*°C
Experiment duration	*t* = 9 h
**Measurement of the thermal constant γ** ${\gamma}_C=\left| \ln \left(\frac{10\hbox{-} 25}{5\hbox{-} 25}\right)\right|\cdot \frac{1}{9}=0.0320\kern0.5em {\mathrm{h}}^{\hbox{-} 1}$ Finally the safety factor *κ* = 1.45 is applied and yields *γ* = 0.0320 1.45 = 0.0463 h^‐1^	

### Step 2: Calculation of the retention time

In step 2, the retention time *t*_*th*_ (in hours) is calculated. Based on the drugs which are transported in the box, the threshold temperature *Θ*_*th*_ has to be selected. The initial temperature is the temperature inside the transport box, before the box is moved to the vehicle *Θ*_*B,i*_ *.*The assumed cabin temperature during the day is used as ambient temperature *Θ*_*A*_. The thermal constant *γ* of the box was calculated in step 1. These values are to be inserted into the following equation

$$ {t}_{t h}=\left| \ln \left(\frac{\Theta_{t h}-{\Theta}_A}{\Theta_{B, i}-{\Theta}_A}\right)\right|\cdot \frac{1}{\gamma} $$

The retention time *t*_*th*_ can be used as estimation if the box can be used to transport the drugs inside the vehicle without risk, that the threshold temperature *Θ*_*th*_ will be exceeded.

**Step 2: Calculation of the retention time**

**Table:**

**Calculation of the retention time** ***t***_***th***_	
Initial temperature	*Θ*_*B,i*_ = 8°C
Threshold temperature (which should not exceeded)	*Θ*_*th*_ = 20 °C
Assumed ambient temperature inside the vehicles	*Θ*_*A*_ = 32 °C
Thermal constant *γ* (calculated in Step1)	*γ =* 0.0463 h^-1^
${t}_{t h}=\left| \ln \left(\frac{20\kern0.5em \hbox{-} \kern0.5em 32}{8\kern0.5em \hbox{-} \kern0.5em 32}\right)\right|\cdot \frac{1}{0.0463}=15\kern0.5em \mathrm{h}$ This means that in a vehicle compartment with an ambient temperature of *Θ*_*A*_ = 32°C the temperature of the drugs inside the box would rise above the threshold temperature *Θ*_*th*_ *=* 20°C after approximately 15 hours. **Attention**: the filling degree of the transport box must be similar to the situation during the measurement of the thermal constant γ (step 1)	
